# Marine Compounds and Cancer: Where Do We Stand?

**DOI:** 10.3390/md13095657

**Published:** 2015-09-02

**Authors:** Sergey A. Dyshlovoy, Friedemann Honecker

**Affiliations:** 1 Department of Oncology, Hematology and Bone Marrow Transplantation with Section Pneumology, Hubertus Wald-Tumorzentrum, University Medical Center Hamburg-Eppendorf, 20246 Hamburg, Germany; E-Mail: dyshlovoy@gmail.com; 2 Laboratory of Marine Natural Products Chemistry, G.B. Elyakov Pacific Institute of Bioorganic Chemistry, Far-East Branch, Russian Academy of Sciences, 690022 Vladivostok, Russian Federation; 3 School of Natural Sciences, Far East Federal University, 690022 Vladivostok, Russian Federation; 4 Tumor and Breast Center ZeTuP St. Gallen, 9006 St. Gallen, Switzerland

In Western countries, cancer is among the most frequent causes of death. Despite striking advances in cancer therapy, there is still an urgent need for new drugs in oncology. Current development favors so called “targeted agents” or drugs that target the immune system, *i.e*., therapeutic antibodies that enhance or facilitate an immune response against tumor cells (also referred to as “checkpoint inhibitors”). However, until recently, roughly 60% of drugs used in hematology and oncology were originally derived from natural sources, and one third of the top-selling agents are either natural agents or derivatives [1]. There is justified hope for the discovery and development of new anticancer agents from the marine environment. Historically, this habitat has proven to be a rich source of potent natural compounds such as alkaloids, steroids, terpenes, macrolides, peptides, and polyketides, among others. Interestingly, marine agents and cancer treatment have had a special relationship from the beginning. One of the first marine-derived compounds, discovered in 1945 that was later developed into a clinically used drug, was spongothymidine [2,3,4], which was the lead compound for the discovery of cytarabine [5]. Until today, cytarabine remains one of the most widely used agents in the treatment of acute myeloid leukemia and relapsed aggressive lymphomas. 

To date, four marine cytotoxic substances, namely cytarabine, trabectidin, eribulin, and monomethylauristatin E (vedotin, derived from dolastatin 10, as part of an antibody–drug conjugate together with brentuximab), have made their way into clinical routine. Many more are in different phases of testing within clinical trials, and a plethora of substances has already been tested for their *in vitro* and *in vivo* activity. 

More and more precise research tools allow the dissection of the molecular mode of action of these cytotoxic substances, thereby uncovering the specific drug targets in cancer cells. This development will potentially blur the edges between “targeted” and “untargeted” therapy in the future, and will hopefully lead to a more targeted use of cancer medicine in general, based on a substance’s molecular mode of action and increasing knowledge regarding specific tumor characteristics on an individual level, *i.e*., by next generation sequencing.

The Special Issue “Marine Compounds and Cancer” (http://www.mdpi.com/journal/marinedrugs/special_issues/marine-compounds-cancer) of the open access journal, *Marine Drugs* (ISSN 1660-3397) was running for two years (2013–2015). It comprises 39 articles in total, a quarter of which are reviews, summarizing the current state of the art in different topics. It covers the full scope from agents with cancer-preventive activity, to novel and previously characterized compounds with anti-cancer activity, both *in vitro* and *in vivo*, and the latest status of clinical development of marine agents used in clinical trials.

The Issue covers a representative selection of different classes of natural or synthetically-derived biomolecules, such as polysaccharides, peptides, lipids, and small-molecule secondary metabolites, isolated from different marine organisms—sponges, ascidia, holothurians, algae, marine fungi, bacteria, and others. Of note, several research articles and reviews submitted to our Special Issue independently from each other, chose the topic of the anticancer properties of the polysaccharide **fucoidan**, indicating that this compound has become one of the most promising and “hot” topics in the field of marine drugs. Likewise, several articles describe different aspects of **trabectedin**. In contrast to fucoidan, trabectedin may be considered to “have made it already”—it is one of first marine-derived anticancer drugs which has been approved for clinical use.

In the following sections, we want to provide a short overview of what the reader will find (and hopefully will also find interesting!) in this Special Issue.

## Review Articles

This Special Issue contains 11 reviews on different topics related to anticancer properties of marine natural compounds. López-Saiz and colleagues from Mexico review the cancer-preventive **lipid compounds from shrimps**, based on their structures and mechanisms of action [6]. Fedorov and colleagues from the Russian Federation discuss structural diversity, biological activity, and the molecular mechanisms of action of **polysaccharides** from different classes of marine organisms, such as macro- and micro-algae, as well as marine fungi, bacteria, and marine animals [7]. Another related review by Kwak from Korea specifically focuses on the polysaccharide **fucoidan**, derived from brown algae. This review provides information on its promising cancer-preventive and anti-cancer activity and mechanism of action both *in vitro* and *in vivo,* and, in addition, summarizes the current status of its preclinical development [8]. A review on the anticancer activity of **small-molecular and polymer compounds isolated from seaweeds** is provided by another Korean group, represented by Moussavou and colleagues [9]. The activity of different compounds, with special emphasis on the polysaccharide fucoidan against colon and breast cancer is reviewed. Kumar and colleagues from Japan contribute a review of the anti-cancer effect of the carotenoid, **fucoxanthin**, including its multiple mechanisms of action *in vitro* and *in vivo* [10]. Newman and Cragg from the USA discuss marine-derived anti-cancer compounds which were under investigation within late pre-clinical development, up to clinical trials in Phases I–III by the end of 2013. Besides cytotoxic (in particular, antibody-drug-conjugates) agents, they also focus on agents that show promising activity against cancer-related pain [11]. Galmarini and colleagues from Italy discuss the mechanisms of action of the marine-derived anti-cancer agents, **trabectedin** and **plitidepsin**. Interestingly, besides direct effects against cancer cells, these substances also exhibit action against the so called tumor microenvironment [12]. Another review on **trabectedin** is provided by Petek and colleagues from the USA. The authors discuss the mechanism of action of the drug, and its activity and development in the treatment of soft tissue sarcomas [13]. Stonik and Fedorov from the Russian Federation provide an up-date on marine cancer preventive **small-molecule compounds**. The literature published between 2003 and 2013 is analyzed, and mechanisms of action are described [14]. Farooqi and colleagues from Pakistan and Taiwan discuss the **ROS modulating effects** of marine-derived compounds, mainly their connection to the regulation of apoptosis and especially autophagy. The effect of both marine- and non-marine derived autophagy modulators (both inducers and inhibitors) in cancer therapy is discussed [15]. Aminin and colleagues from the Russian Federation summarize recent findings on anticancer activity of **triterpene glycosides** derived from sea cucumbers, and their molecular mechanisms of action [16].

## Research Articles

Recent findings in chemistry and biology of anticancer compounds isolated from marine animals can be found in this Special Issue in 28 original research articles. Yang and colleagues from China report the apoptosis-inducing activity of the polysaccharide **fucoidan**, isolated from *Undaria pinnatifida,* in human hepatocellular carcinoma cells *in vitro*. A ROS-mediated mitochondrial pathway is suggested to be critical for this process [17]. Another comparative study analyzing cytotoxic (apoptosis-inducing) effects of native **fucoidan** and **fucoidan lipid nanoparticles** on osteosarcoma cells *in vitro* and *in vivo* is provided by Kimura and colleagues from Japan [18]. Zovco and colleagues from Slovenia, Sweden, USA, and Italy show that **APS8** (a synthetic analog of 3-alkylpyridinium polymer from the marine sponge *Reniera sarai*) inhibits the growth and induces apoptosis in non-small cell lung cancer cells, while normal lung fibroblasts are not affected. APS8 also reduces anti-apoptotic and pro-proliferative effects of nicotine [19]. Li and colleagues from China apply a global proteome screening approach to identify the molecular targets of **sinulariolide** (a compound from the cultured soft coral *Sinularia flexibilis*) in melanoma cells. The results suggest that sinulariolide-induced apoptosis might be related to activation of caspase cascades and mitochondria dysfunction pathways [20]. Li and colleagues from China isolated five new anthranilic acid derivatives, **penipacids A**–**E**, from the marine sediment-derived fungus *Penicillium paneum* SD-44. Penipacids A and E are cytotoxic to human colon cancer RKO cells [21]. Walsh and colleagues from the USA describe caspase-dependent and mitochondrial pathway-related apoptosis in leukemia cells in response to treatment with **epigonal conditioned medium** from the bonnethead shark, *Sphyrna tiburo* [22]. Su and colleagues from Taiwan and Egypt investigate the mechanism underlying the cytotoxic action of **(1′*R*,5′*S*,6′*S*)-2-(3′,5′-dibromo-1′,6′-dihydroxy-4′-oxocyclohex-2′-enyl) acetonitrile**, previously isolated from the sponge *Pseudoceratina* sp., in leukemia cells. The effect is associated with mitochondrial dysfunction-dependent apoptosis, and the process is mediated by oxidative stress, induced through inhibition of IKK/NFkB and activation of PI3K/Akt pathways [23]. Ishikawa and colleagues from Japan examined anticancer activity and the mode of action of **hippuristanol** isolated from the Okinawan coral, *Isis hippuris,* on primary effusion lymphoma cells. The authors show that this compound induces cell cycle arrest and apoptosis and suppresses several pro-survival pathways *in vitro,* and growth and invasiveness *in vivo* [24]. Esmaeelian and colleagues from Australia report the bioassay guided isolation of semi-pure **tyrindoleninone** and **6-bromoisatin** from an egg mass extract of the marine gastropod, *Dicathais orbita*. The compounds induce apoptosis and G2/M cell cycle arrest in colorectal cancer cells [25]. In a subsequent manuscript, *in vivo* activity of the synthetic 6-bromoisatin in a mouse model of colorectal cancer cells was reported from the same group [26]. Király and colleagues from Hungary and Spain investigated the effect of hypoxia on the efficiency of the substance **elisidepsin** (**Irvalec^®^**). The authors show that hypoxia significantly inhibits the anti-tumor effect of elisidepsin, and they show that this is mediated by reduced levels of 2-hydroxy lipids in the cancer cell membranes [27]. Li and colleagues from Korea and China investigated the *in vitro* anti-cancer activity of the alkaloid **fumigaclavine C**, isolated from the marine-derived fungus *Aspergillus fumigatus*, using a breast cancer model. They show regulation of several apoptosis- and proliferation/migration-related proteins, as well as inhibition of the NFkB pathway [28]. Pereira and colleagues from Portugal investigated the effect of an extract of the sea star *Marthasterias glacialis* L. on human breast cancer and neuroblastoma cells. **Palmitic acid** and **ergosta-7,22-dien-3-ol** are the main components of the extract responsible for the anti-cancer activity [29]. García-Caballero and colleagues from Spain report *in vitro* and *in vivo* anti-angiogenetic activity of the **pyrrolidinedione AD0157**, isolated from the marine fungus *Paraconiothyrium* sp. They attribute the activity to an inhibition of the Akt signaling pathway, which could place the substance in the group of targeted agents in the future [30]. Hamilton from Austria investigated the cytotoxic effect of the marine spongean alkaloid **fascaplysin** in small cell lung cancer cells. The mechanism of action appears to be multi-factorial, and can be related to alteration of topoisomerase I activity, impaired integrity of DNA, and ROS generation [31]. Fuwa and colleagues from Japan report the induction of both apoptotic and non-apoptotic cell death by **(−)-8,9-dehydroneopeltolide**—a synthetic analog of the marine macrolide (+)-neopeltolide—under cellular stress conditions. The induction of non-apoptotic cell death was explained by an intracellular ATP depletion induced by the compound [32]. Akl and colleagues from USA, Jordan and Kuwait describe the ability of **araguspongine C** (a macrocyclic oxaquinolizidine alkaloid isolated from the marine sponge *Xestospongia* sp.) to act as a tyrosine kinases receptor inhibitor, providing even more evidence that naturally occurring substances might act like targeted agents. Interestingly, this inhibitory effect induces autophagic cell death in breast cancer cells [33]. Kasper and colleagues from Germany report the results of a Phase I study of gemcitabine in combination with **trabectedin** in advanced soft tissue sarcomas. The study had to be stopped due to excessive toxicity, which adds important clinical information for further development of combination therapies in patients with advanced and/or metastatic leiomyosarcoma or liposarcoma [34]. Kwon and colleagues from Korea present data on the synthesis and *in vitro* anti-cancer activity of derivatives of **pachastrissamine**—a marine sphingolipid initially isolated from the marine sponge *Pachastrissa* sp. An enyne/diene-ene metathesis reaction was used as the key step of the synthesis. One of the derivatives synthesized exhibits more potent sphingosine kinases inhibitory activity in comparison with the mother, pachastrissamine [35]. Wu and colleagues from China describe the anticancer activity of the synthetical **bis-(2,3-Dibromo-4,5-dihydroxy-phenyl)-methane**, initially isolated from marine algae *Rhodomelaceae confervoides*. The compound inhibits proliferation, migration, and invasion of hepatocellular carcinoma cells, which is explained by modulation of the β1-integrin/FAK signaling pathway [36]. Kim and colleagues from Korea investigated the anticancer effect of **(1*S*,2*S*,3*E*,7*E*,11*E*)-3,7,11,15-cembratetraen-17,2-olide**, isolated from the marine soft coral *Lobophytum* sp., in fluorouracil-resistant human colon cancer cells. They suggest that the mechanism of action is related to the activation of TGF-β signaling [37]. Song and colleagues from China report the isolation of two new and three previously known C-glycoside **angucyclines** from an extract of the deep-sea sediment bacteria *Streptomyces* sp. Several of these compounds exhibit strong cytotoxic activity in human cancer cells, comparable to cisplatin [38]. Morgan and colleagues from the USA studied bioactivity of the lipopeptide **kalkitoxin**, which was previously isolated from the marine cyanobacteria, *Moorea producens* (*Lyngbya majuscula*). The compound inhibits angiogenesis, disrupts cellular hypoxic signaling, and blocks mitochondrial electron transport in human cancer cells [39]. Wang and colleagues from China describe the ability of the marine anthraquinone derivative **SZ-685C** (isolated from the fungus *Halorosellinia* sp.) to induce apoptosis of primary human nonfunctioning pituitary adenoma cells. The effect is explained by the ability to inhibit the Akt pathway [40]. Kim and colleagues from Korea studied the ability of **dieckol** (or **2,7″-phloroglucinol-6,6′-bieckol**) isolated from brown algae *Ecklonia cava* to inhibit the migration of human breast cancer cells. Expression of several metastasis-related genes is regulated in cancer cells treated with the compound [41]. Zhang and colleagues from China, Saudi Arabia, and the USA report the ability of esters of the **sipholenol A**—a sipholane triterpenoid from the marine sponge *Siphonochalina siphonella*—to specifically reverse P-glycoprotein-mediated multidrug resistance, and *in vitro* and *in silico* analyses are presented [42]. Chen and colleagues from Canada and China describe the suppressive effect of **xyloketal B**, isolated from the mangrove fungus *Xylaria* sp., on glioblastoma cell proliferation and migration. Another “targeted” action via inhibition of TRPM7-regulated PI3K/Akt and MEK/ERK signaling is suggested as the underlying mechanism [43]. Finally, Perina and colleagues from Croatia, Israel, and the United Kingdom studied the role of the highly conservative ***FAU* gene** from the sponge *Suberites domuncula*. The authors describe a pro-apoptotic activity of both spongean and human FAU proteins. These findings provide an opportunity to use pre-bilaterian animals as a simpler model for studying complex interactions in human cancerogenesis [44].

In summary, this Issue covers a broad spectrum of excellent work recently done in the field of marine compounds and cancer. It will serve as a valuable source for current state-of-the-art knowledge in the field, comprising both exciting new research and up-to-date reviews. The guest editors are thankful to all scientists currently working in diverse research institutes, universities, and commercial companies all over the world, namely in Australia, Austria, Canada, China, Croatia, Egypt, Germany, Hungary, Israel, Italy, Japan, Jordan, Korea, Kuwait, Mexico, Pakistan, Portugal, the Russian Federation, Saudi Arabia, Slovenia, Spain, Sweden, Taiwan, the United Kingdom and the USA, who contributed to the success of our Special Issue “Marine Compounds and Cancer”. We are all looking forward to new exciting discoveries!

Dr. Sergey A. Dyshlovoy and Dr. Friedemann Honecker
Guest Editors, Special Issue “Marine Compounds and Cancer”,
Editorial Board Members, *Marine Drugs*

## References

[1] Singh R., Sharma M., Joshi P., Rawat D.S. (2008). Clinical status of anti-cancer agents derived from marine sources. Anticancer Agents Med. Chem..

[2] Bergmann W., Burke D.C. (1956). Contributions to the Study of Marine Products. XL. The Nucleosides of Sponges.1 IV. Spongosine 2. J. Org. Chem..

[3] Bergmann W., Feeney R.J. (1951). Contributions to the study of marine products. XXXII. The nucleosides of spongies. I.. J. Org. Chem..

[4] Bergmann W., Stempien M.F. (1957). Contributions to the Study of Marine Products. XLIII. The Nucleosides of Sponges. V. The Synthesis of Spongosine 1. J. Org. Chem..

[5] Stonik V. (2009). Marine natural products: A way to new drugs. Acta Naturae.

[6] López-Saiz C.-M., Suárez-Jiménez G.-M., Plascencia-Jatomea M., Burgos-Hernández A. (2013). Shrimp Lipids: A Source of Cancer Chemopreventive Compounds. Mar. Drugs.

[7] Fedorov S., Ermakova S., Zvyagintseva T., Stonik V. (2013). Anticancer and Cancer Preventive Properties of Marine Polysaccharides: Some Results and Prospects. Mar. Drugs.

[8] Kwak J.-Y. (2014). Fucoidan as a Marine Anticancer Agent in Preclinical Development. Mar. Drugs.

[9] Moussavou G., Kwak D., Obiang-Obonou B., Maranguy C., Dinzouna-Boutamba S.-D., Lee D., Pissibanganga O., Ko K., Seo J., Choo Y. (2014). Anticancer Effects of Different Seaweeds on Human Colon and Breast Cancers. Mar. Drugs.

[10] Kumar S., Hosokawa M., Miyashita K. (2013). Fucoxanthin: A Marine Carotenoid Exerting Anti-Cancer Effects by Affecting Multiple Mechanisms. Mar. Drugs.

[11] Newman D., Cragg G. (2014). Marine-Sourced Anti-Cancer and Cancer Pain Control Agents in Clinical and Late Preclinical Development. Mar. Drugs.

[12] Galmarini C., Incalci M., Allavena P. (2014). Trabectedin and Plitidepsin: Drugs from the Sea that Strike the Tumor Microenvironment. Mar. Drugs.

[13] Petek B., Loggers E., Pollack S., Jones R. (2015). Trabectedin in Soft Tissue Sarcomas. Mar. Drugs.

[14] Stonik V., Fedorov S. (2014). Marine Low Molecular Weight Natural Products as Potential Cancer Preventive Compounds. Mar. Drugs.

[15] Farooqi A., Fayyaz S., Hou M.-F., Li K.-T., Tang J.-Y., Chang H.-W. (2014). Reactive Oxygen Species and Autophagy Modulation in Non-Marine Drugs and Marine Drugs. Mar. Drugs.

[16] Aminin D., Menchinskaya E., Pisliagin E., Silchenko A., Avilov S., Kalinin V. (2015). Anticancer Activity of Sea Cucumber Triterpene Glycosides. Mar. Drugs.

[17] Yang L., Wang P., Wang H., Li Q., Teng H., Liu Z., Yang W., Hou L., Zou X. (2013). Fucoidan Derived from Undaria pinnatifida Induces Apoptosis in Human Hepatocellular Carcinoma SMMC-7721 Cells via the ROS-Mediated Mitochondrial Pathway. Mar. Drugs.

[18] Kimura R., Rokkaku T., Takeda S., Senba M., Mori N. (2013). Cytotoxic Effects of Fucoidan Nanoparticles against Osteosarcoma. Mar. Drugs.

[19] Zovko A., Viktorsson K., Lewensohn R., Kološa K., Filipič M., Xing H., Kem W., Paleari L., Turk T. (2013). APS8, a Polymeric Alkylpyridinium Salt Blocks α7 nAChR and Induces Apoptosis in Non-Small Cell Lung Carcinoma. Mar. Drugs.

[20] Li H.-H., Su J.-H., Chiu C.-C., Lin J.-J., Yang Z.-Y., Hwang W.-I., Chen Y.-K., Lo Y.-H., Wu Y.-J. (2013). Proteomic Investigation of the Sinulariolide-Treated Melanoma Cells A375: Effects on the Cell Apoptosis through Mitochondrial-Related Pathway and Activation of Caspase Cascade. Mar. Drugs.

[21] Li C.-S., Li X.-M., Gao S.-S., Lu Y.-H., Wang B.-G. (2013). Cytotoxic Anthranilic Acid Derivatives from Deep Sea Sediment-Derived Fungus *Penicillium paneum* SD-44. Mar. Drugs.

[22] Walsh C., Luer C., Yordy J., Cantu T., Miedema J., Leggett S., Leigh B., Adams P., Ciesla M., Bennett C., Bodine A. (2013). Epigonal Conditioned Media from Bonnethead Shark, *Sphyrna tiburo*, Induces Apoptosis in a T-Cell Leukemia Cell Line, Jurkat E6-1. Mar. Drugs.

[23] Su J.-H., Chen Y.-C., El-Shazly M., Du Y.-C., Su C.-W., Tsao C.-W., Liu L.-L., Chou Y., Chang W.-B., Su Y.-D. (2013). Towards the Small and the Beautiful: A Small Dibromotyrosine Derivative from *Pseudoceratina* sp. Sponge Exhibits Potent Apoptotic Effect through Targeting IKK/NFκB Signaling Pathway. Mar. Drugs.

[24] Ishikawa C., Tanaka J., Katano H., Senba M., Mori N. (2013). Hippuristanol Reduces the Viability of Primary Effusion Lymphoma Cells both *in Vitro* and *in Vivo*. Mar. Drugs.

[25] Esmaeelian B., Benkendorff K., Johnston M., Abbott C. (2013). Purified Brominated Indole Derivatives from Dicathais orbita Induce Apoptosis and Cell Cycle Arrest in Colorectal Cancer Cell Lines. Mar. Drugs.

[26] Esmaeelian B., Abbott C., Le Leu R., Benkendorff K. (2013). 6-Bromoisatin Found in Muricid Mollusc Extracts Inhibits Colon Cancer Cell Proliferation and Induces Apoptosis, Preventing Early Stage Tumor Formation in a Colorectal Cancer Rodent Model. Mar. Drugs.

[27] Király A., Váradi T., Hajdu T., Rühl R., Galmarini C., Szöllősi J., Nagy P. (2013). Hypoxia Reduces the Efficiency of Elisidepsin by Inhibiting Hydroxylation and Altering the Structure of Lipid Rafts. Mar. Drugs.

[28] Li Y.-X., Himaya S. W. A., Dewapriya P., Zhang C., Kim S.-K. (2013). Fumigaclavine C from a Marine-Derived Fungus Aspergillus Fumigatus Induces Apoptosis in MCF-7 Breast Cancer Cells. Mar. Drugs.

[29] Pereira D., Correia-da-Silva G., Valentão P., Teixeira N., Andrade P. (2013). Palmitic Acid and Ergosta-7,22-dien-3-ol Contribute to the Apoptotic Effect and Cell Cycle Arrest of an Extract from *Marthasterias glacialis* L. in Neuroblastoma Cells. Mar. Drugs.

[30] García-Caballero M., Cañedo L., Fernández-Medarde A., Medina M., Quesada A. (2014). The Marine Fungal Metabolite, AD0157, Inhibits Angiogenesis by Targeting the Akt Signaling Pathway. Mar. Drugs.

[31] Hamilton G. (2014). Cytotoxic Effects of Fascaplysin against Small Cell Lung Cancer Cell Lines. Mar. Drugs.

[32] Fuwa H., Sato M., Sasaki M. (2014). Programmed Cell Death Induced by (−)-8,9-Dehydroneopeltolide in Human Promyelocytic Leukemia HL-60 Cells under Energy Stress Conditions. Mar. Drugs.

[33] Akl M., Ayoub N., Ebrahim H., Mohyeldin M., Orabi K., Foudah A., Sayed K. (2015). Araguspongine C Induces Autophagic Death in Breast Cancer Cells through Suppression of c-Met and HER2 Receptor Tyrosine Kinase Signaling. Mar. Drugs.

[34] Kasper B., Reichardt P., Pink D., Sommer M., Mathew M., Rauch G., Hohenberger P. (2015). Combination of Trabectedin and Gemcitabine for Advanced Soft Tissue Sarcomas: Results of a Phase I Dose Escalating Trial of the German Interdisciplinary Sarcoma Group (GISG). Mar. Drugs.

[35] Kwon Y., Song J., Bae H., Kim W.-J., Lee J.-Y., Han G.-H., Lee S., Kim S. (2015). Synthesis and Biological Evaluation of Carbocyclic Analogues of Pachastrissamine. Mar. Drugs.

[36] Wu N., Luo J., Jiang B., Wang L., Wang S., Wang C., Fu C., Li J., Shi D. (2015). Marine Bromophenol Bis(2,3-Dibromo-4,5-dihydroxy-phenyl)-methane Inhibits the Proliferation, Migration, and Invasion of Hepatocellular Carcinoma Cells via Modulating β1-Integrin/FAK Signaling. Mar. Drugs.

[37] Kim E.-J., Kang J.-I., Kwak J.-W., Jeon C.-H., Tung N.-H., Kim Y.-H., Choi C.-H., Hyun J.-W., Koh Y.-S., Yoo E.-S. (2015). The Anticancer Effect of (1*S*,2*S*,3*E*,7*E*,11*E*)-3,7,11,15-Cembratetraen-17,2-olide(LS-1) through the Activation of TGF-β Signaling in SNU-C5/5-FU, Fluorouracil-Resistant Human Colon Cancer Cells. Mar. Drugs.

[38] Song Y., Liu G., Li J., Huang H., Zhang X., Zhang H., Ju J. (2015). Cytotoxic and Antibacterial Angucycline- and Prodigiosin-Analogues from the Deep-Sea Derived *Streptomyces* sp. SCSIO 11594. Mar. Drugs.

[39] Morgan J., Liu Y., Coothankandaswamy V., Mahdi F., Jekabsons M., Gerwick W., Valeriote F., Zhou Y.-D., Nagle D. (2015). Kalkitoxin Inhibits Angiogenesis, Disrupts Cellular Hypoxic Signaling, and Blocks Mitochondrial Electron Transport in Tumor Cells. Mar. Drugs.

[40] Wang X., Tan T., Mao Z.-G., Lei N., Wang Z.-M., Hu B., Chen Z.-Y., She Z.-G., Zhu Y.-H., Wang H.-J. (2015). The Marine Metabolite SZ-685C Induces Apoptosis in Primary Human Nonfunctioning Pituitary Adenoma Cells by Inhibition of the Akt Pathway *in Vitro*. Mar. Drugs.

[41] Kim E.-K., Tang Y., Kim Y.-S., Hwang J.-W., Choi E.-J., Lee J.-H., Lee S.-H., Jeon Y.-J., Park P.-J. (2015). First Evidence that *Ecklonia cava*-Derived Dieckol Attenuates MCF-7 Human Breast Carcinoma Cell Migration. Mar. Drugs.

[42] Zhang Y., Zhang Y.-K., Wang Y.-J., Vispute S., Jain S., Chen Y., Li J., Youssef D., Sayed K., Chen Z.-S. (2015). Esters of the Marine-Derived Triterpene Sipholenol A Reverse P-GP-Mediated Drug Resistance. Mar. Drugs.

[43] Chen W.-L., Turlova E., Sun C., Kim J.-S., Huang S., Zhong X., Guan Y.-Y., Wang G.-L., Rutka J., Feng Z.-P. (2015). Xyloketal B Suppresses Glioblastoma Cell Proliferation and Migration *in Vitro* through Inhibiting TRPM7-Regulated PI3K/Akt and MEK/ERK Signaling Pathways. Mar. Drugs.

[44] Perina D., Korolija M., Hadžija M., Grbeša I., Belužić R., Imešek M., Morrow C., Marjanović M., Bakran-Petricioli T., Mikoč A. (2015). Functional and Structural Characterization of FAU Gene/Protein from Marine Sponge *Suberites domuncula*. Mar. Drugs.

